# Patterns of postural deformity in non-ambulant people with cerebral palsy: what is the relationship between the direction of scoliosis, direction of pelvic obliquity, direction of windswept hip deformity and side of hip dislocation?

**DOI:** 10.1177/0269215507080121

**Published:** 2008

**Authors:** David Porter, Shona Michael, Craig Kirkwood

**Affiliations:** School of Health & Social Care, Oxford Brookes University, Oxford; Medical Physics & Engineering, Leeds University Teaching Hospitals NHS Trust, Leeds; Tayside Assistive Technology Service, Acute Services Division NHS Tayside, Dundee, UK

## Abstract

**Objective**: To investigate: (a) associations between the direction of scoliosis, direction of pelvic obliquity, direction of windswept deformity and side of hip subluxation/dislocation in non-ambulant people with cerebral palsy; and (b) the lateral distribution of these postural asymmetries.

**Design**: Cross-sectional observational study.

**Setting**: Posture management services in three centres in the UK.

**Subjects**: Non-ambulant people at level five on the gross motor function classification system for cerebral palsy.

**Main measures**: Direction of pelvic obliquity and lateral spinal curvature determined from physical examination, direction of windswept hip deformity derived from range of hip abduction/adduction, and presence/side of unilateral hip subluxation defined by hip migration percentage.

**Results**: A total of 747 participants were included in the study, aged 6–80 years (median 18 years 10 months). Associations between the direction of scoliosis and direction of pelvic obliquity, and between the direction of windswept hip deformity and side hip subluxation/dislocation were confirmed. A significant association was also seen between the direction of scoliosis and the direction of the windswept hip deformity (*P* < 0.001) such that the convexity of the lateral spinal curve was more likely to be opposite to the direction of windsweeping. Furthermore, significantly more windswept deformities to the right (*P* = 0.007), hips subluxed on the left (*P* = 0.002) and lateral lumbar/lower thoracic spinal curves convex to the left (*P* = 0.03) were observed.

**Conclusions**: The individual asymmetrical postural deformities are not unrelated in terms of direction and not equally distributed to the left/right. A pattern of postural deformity was observed.

## Introduction

People with cerebral palsy who are non-ambulant are particularly vulnerable to the development of contractures and postural deformity, which are often progressive despite the fact that the underlying pathology is static.^1^,^2^ The asymmetrical postural configurations or ‘deformities’ that can arise include scoliosis, pelvic obliquity and hip subluxation/dislocation. Windswept hips deformity is also often experienced where there is an abduction contracture of one hip and an adduction contracture of the opposite hip.^3^,^4^ These postural deformities can result in secondary problems such as pain, loss of ability and independence, pressure ulcers, cardiovascular and respiratory problems, swallowing difficulties and sleep disturbance, all of which are likely to have a significant effect on quality of life.^5–9^

Individual components of asymmetrical postural deformity tend to be considered and studied separately, however, a clear understanding of the relationship between them is essential if patterns of deformity are to be predicted and early postural management strategies implemented. Previous research has reported that scoliosis is often accompanied by pelvic obliquity, windsweeping and/or hip dislocation^3–5^,^8^,^10–12^ although evidence of any relationship has been contradictory and inconclusive.

Letts *et al*.^3^ studied 22 teenagers with cerebral palsy and a ‘triad’ of asymmetric hip deformity, pelvic obliquity and scoliosis. From a series of radiographs it was reported that the spinal curvature was convex away from the dislocated hip for 17 participants. The authors concluded that the more subluxed hip was likely to occur on the elevated side of the pelvis because, in the presence of pelvic obliquity, the acetabulum on the high side becomes more vertical which uncovers the hip and leads to subluxation. However this was not supported by the findings in several subsequent studies by other researchers.^4^,^10–12^

Lonstein and Beck^10^ found no evidence of association between direction of pelvic obliquity and side of hip subluxation/dislocation based on an analysis of radiographs of a subgroup of 77 people from a larger cross-sectional study of people with cerebral palsy seen in their service. In addition, no association was seen between direction of windsweeping and pelvic obliquity for another subgroup of 53 people. The authors concluded that no relationship existed between hip subluxation, pelvic obliquity, windswept deformity and scoliosis and suggested that muscle imbalance around the hip, rather than pelvic obliquity, was the cause of the hip subluxation or dislocation.

In a study of prevalence of neuromuscular scolioisis Madigan and Wallace^11^ reported on a subgroup of 36 quadriplegic participants who had both windswept hip deformity and scoliosis, indicating that the femurs pointed towards the concavity of the scoliosis in 22 cases and toward the convexity in 14 cases. However, applying a Pearson's chi-squared test to the author's original data indicates no statistically significant association.

More recently Young *et al*.^4^ carried out a retrospective/cross-sectional study involving physical examination of people with spastic quadriplegic cerebral palsy. Although they found evidence of a relationship between tonal asymmetry and direction of windswept deformity in a subgroup of 33 participants, with the hips tending to windsweep toward the side of lower tone, they found no relationship between direction of tone and direction of lateral spinal curvature in another subgroup of 22 participants. Furthermore, no relationship was found between direction of windswept deformity and direction of scoliosis in the 26 participants who demonstrated both deformities.

When Abel *et al*.^12^ carried out a small prospective cohort study involving 37 people with cerebral palsy and windswept deformity they hypothesized that based on the findings of Letts *et al*.^3^ the more subluxed hip would be opposite the scoliosis apex and ipsilateral to the high side of the pelvis. However, this could not be substantiated and the authors actually found that hip subluxation strongly correlated with the degree of femoral adduction and weakly with the magnitude of suprapelvic obliquity.

In all of the above studies the numbers of participants with both relevant components of postural deformity were too low to give the studies sufficient statistical power to test for evidence of association. Therefore the main aim of this study was to investigate the association between the direction of asymmetrical postural deformities in non ambulant people with cerebral palsy using a study population of statistically appropriate size. The study concentrated on the direction and not the degree of asymmetrical deformity. The specific null hypotheses tested assumed no relationship between:

- direction of lateral spinal curvature and direction of pelvic obliquity;
- direction of windswept deformity and side of hip subluxation/dislocation;
- direction of lateral spinal curvature and direction of windswept deformity; and
- direction of pelvic obliquity and side of hip subluxation/dislocation.

A secondary aim of the study was to analyse the lateral distribution of these asymmetrical postural deformities. Therefore a further series of null hypotheses were tested which assumed equal proportions of the lateral postural deformities deviating from a neutral position to the left and right.

## Method

### Subjects

All subjects had bilateral cerebral palsy resulting in them being non-ambulant and at level five on the gross motor function classification system for cerebral palsy (i.e. lacking independence in basic antigravity postural control).^13^ More specifically, they had a Chailey sitting ability of level 1 (could not be placed in upright sitting position), level 2 (could be placed in a sitting position but unable to maintain it) or level 3 (could maintain position but only if he/she did not move at all).^14^

The study population consisted of all patients meeting the above criteria and undergoing routine physical assessment in the posture management clinics at three centres in the UK between 1993 and 2004. Selection was avoided in order to minimize potential bias. The three centres (Tayside Orthopaedic and Rehabilitation Technology Centre; the West of Scotland Mobility and Rehabilitation Centre; and the Oxford Centre for Enablement) were used as they had adopted similar physical/postural assessment procedures and documentation.^15^

Sample size calculations were undertaken^16^. Without evidence to the contrary, a 50% frequency of a deformity occurring to the left or right was anticipated. In order to detect a 10% deviation in observed frequency with 5% significance and 80% power it was estimated that approximately 750 participants would be required to produce robust findings.

### Assessment procedure

Participants were initially positioned in a sitting posture on the edge of an examination couch. Support for the trunk was provided by one of the assessors in order to maintain a stable sitting position. The posterior processes of the vertebrae were palpated and marked with small black stickers. If any lateral curvature of the spine was observed the assessor noted the direction of the main curve and that of any other minor curves. The assessor also noted the level of the apex of each curve. Still sitting, with head and trunk balanced over the pelvis, the anterior superior iliac spines were located and used to estimate the orientation of the pelvis in the coronal plane. The assessor established whether the left or right anterior superior iliac spine was lower, or if both were level.

Hip abduction/adduction range was used as an indicator of the direction of windsweeping. It was measured with the participant lying in a supine position with the hip under examination, and also the knee on the ipsilateral side, flexed to 90° (or as near 90° as could be achieved). Flexion was preferred because it approximated better to the posture of the hip during sitting and for infants who have restricted hip movement, abduction is often only possible in the flexed position.^17^,^18^ The contralateral hip and knee were left in the neutral (extended) position or very slight flexed. The neutral position was defined with the axis of the thigh lying on a line perpendicular to a transverse line across the anterior superior iliac spines.^17^ Abduction and adduction were measured as the angular displacements of the thigh from this neutral axis with the limits of range taken as the points at which the pelvis began to rotate anteriorly on the contralateral and ipsilateral side respectively. In order to ensure consistent recording and interpretation of joint range data, the Neutral-0 method of notation was used.^17^

The status of both left and right hip joint was recorded in order to indicate whether there was any unilateral or bilateral subluxation (or dislocation). This was done by referring to the latest orthopaedic review and routine surveillance X-ray images of the hips and pelvis usually carried out annually for this population given their high risk of experiencing hip deformity.^2^ Hip status was defined by hip migration percentage (the proportion of the femoral head lying beyond the lateral margin of the acetabulum)^19–21^ taken from X-ray images showing an anteroposterior view of the hip and pelvis with the subject lying supine. The hip was regarded as ‘subluxed’ if the migration index was 33% or greater.^21^,^22^

### Data analysis

A variable was generated to indicate the presence and direction of a windswept hip pattern based on asymmetry of hip abduction/adduction range. The variable was calculated using the following formula with abduction regarded as a positive angle and adduction a negative angle.

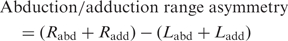

where *R*_abd_ is range of abduction at the right hip, *R*_add_ is range of adduction at the right hip, *L*_abd_ is range of abduction at the left hip and *L*_add_ is range of adduction at the left hip.

A positive value for the above variable was indicative of windsweeping to the right and a negative value windsweeping to the left. However, due to the potential margin of error in measurement of abduction/adduction, thresholds of >10° and <−10° were used to indicate windsweeping right and left respectively.^15^

Analysis was carried out using SPSS. The strength of the evidence against the null hypothesis of no association between the direction of deformities was tested using a Pearson chi-squared test.^16^ The lateral distribution of deformities was examined using a one-sample chi-square test. The direction of a single lateral spinal curve, or lower of a double curve configuration, was used in the analysis when testing association between spinal curvature and other variables. Tests of association involving windswept direction as a categorical variable were complemented by carrying out analysis of variance of the continuous abduction/adduction joint range variable.^16^

Testing was restricted to the predetermined hypotheses. Subgroup analysis was only carried out where there was a logical basis for separate analysis (pre-defined within the protocol) (e.g. analysis of spinal curves categorized by the level of the apex of the curve).

## Results

Out of 747 participants 388 (51.9%) were male and 359 (48.1%) were female. Ages ranged from 6 years to 80 years as shown in Table 1 although the median age at 18 years and 10 months was closer to the younger end of the range. As defined by the inclusion criteria, not only could subjects not walk but they also lacked independence in antigravity postural control, resulting in Chailey levels of sitting ability as shown in Table 2.

**Table 1:** Age of subjects

Age range (years)	Number	Percentage
6–10	135	18.1
11–15	143	19.1
16–20	133	17.0
21–25	68	9.1
26–30	56	7.5
31–35	61	8.2
36–40	40	5.4
41+	111	14.9
Total	747	100

**Table 2:** Sitting ability of subjects

Chailey level of sitting ability	Number	Percentage
1	309	41.4
2	271	36.3
3	169	22.3
Total	747	100

Lateral spinal curves were recorded for 690 participants, pelvic obliquity for 644 and windswept hip deformity for 417. There were 258 left hips and 192 right hips subluxed or dislocated with a total of 233 unilateral and 89 bilateral hip subluxation/dislocations. The lateral distribution of the asymmetrical postural deformities are shown in Table 3. This table also shows the lateral spinal curves categorized by the level of the apex of the curve and by configuration with the majority (518, 75.1%) being single curves.

**Table 3:** Direction of asymmetrical postural deformities

	Direction of asymmetrical postural deformity			
	
	Left	Right	N/A	Total
Individuals with lateral spinal curve (convex to the left/right)	364	326	57	747
Level of apex of spinal curve				
Higher thoracic	3	7	–	10
Mid thoracic	208	201	–	409
Lower thoracic	112	86	–	198
Lumbar	41	32	–	73
Configuration of spinal curve				
Single curve	272	246	–	518
Double curve	92	80	–	172
Total lateral spinal curves (convex to the left/right)	444	418	–	862
Individuals with pelvic obliquity (ASIS lower on left/right)	332	312	103	747
Individuals with windswept hip pattern (toward left/right)	181	236	330	747
Individuals with unilateral hip subluxation/dislocation (left/right)	140	93	514	747

There was no significant difference between the total number of people with an oblique pelvis lower on the left (95% confidence interval (CI) 47.6% to 55.6%) compared with lower on the right.

Also, although more people had lateral curves convex to the left rather than the right (95% CI 49.2% to 56.4%), and there were more convex left curves in total when counting both of the double curves (95% CI 48.0% to 54.8%), these differences were also not statistically significant (*P* = 0.15 and *P* = 0.39, respectively). However, when looking specifically at the 271 individuals with spinal curves at lumbar or lower thoracic level, there was a statistically significant disproportion of curves which were convex to the left (95% CI 50.6% to 62.4%; *P* = 0.033).

Furthermore, a significantly greater number of people with windswept hip patterns were windswept to the right (95% CI 51.8% to 61.4%; *P* = 0.007) and there was a significant disproportion of participants who had unilateral subluxation of the left rather than the right hip (95% CI 53.7% to 66.5%; *P* = 0.002).

Table 4 shows a cross-tabulation of the direction of lateral spinal curvature (the single curve configurations or the lower of the double configuration) and the direction of pelvic obliquity for the 638 people with both postural asymmetries. This provided evidence to suggest that the direction of the lateral curvature and pelvic obliquity were not independent (χ^2^ = 545.75; *P* < 0.001). Similar evidence was seen when looking specifically at people with lumbar, lower thoracic and mid-thoracic level spinal curves (all *P* < 0.001).

**Table 4:** Cross-tabulation of pelvic obliquity and lateral spinal curvature

	Direction of lateral spinal curve		
	
	Convex left	Convex right	Total
Pelvic obliquity			
Lower on left	323	8	331
Lower on right	16	291	307
Total	339	299	638

The direction of windsweeping and the side of hip anomaly for the 166 people who had both an asymmetrical range of adduction/abduction and a unilaterally subluxed or dislocated hip is shown in Table 5. Significantly more hips were found to be subluxed on the left when the windswept pattern was to the right, and vice versa (χ^2^ = 89.88, *P* < 0.001). An ANOVA, taking the abduction/adduction range index as the dependent variable and hip status as the factor, also indicated a significant variance between groups (*F* = 172.50; *P* < 0.001).

**Table 5:** Cross-tabulation of windswept deformity and hip subluxation

Windswept deformity	Hip subluxation/dislocation		
	
	Left	Right	Total
Left	9	55	64
Right	90	12	102
Total	99	67	166

Table 6 shows the direction of the lateral curvature of the spine and the direction of windsweeping for 386 people for whom both postural asymmetries were recorded. A significantly larger proportion of people with a windswept pattern to the right appeared to have a lateral spinal curve convex to the left, and vice versa (χ^2^ = 22.19, *P* < 0.001). The evidence to suggest that these two variables were not independent was strongest for participants who had mid-thoracic spinal curves (*P* < 0.001) and for all participants with single curve configurations (*P* < 0.001). This relationship was supported by an ANOVA, taking the abduction/adduction asymmetry as the dependent variable and direction of convexity of the lateral spinal curve as the factor, which indicated a significant variance between groups (*F* = 30.14; *P* < 0.001).

**Table 6:** Cross-tabulation of windswept deformity and lateral spinal curvature

Windswept deformity	Lateral spinal curve		
	
	Convex left	Convex right	Total
Left	66	98	164
Right	143	79	222
Total	209	177	386

Further chi-squared tests were carried out between the direction of lateral spinal curvature and windswept hip deformity with participants divided into categories according to their age at assessment. The strength of evidence against the null hypothesis of independence was significant (*P* < 0.005) for age groups 11–20, 21–30 and 31–40 years, but it was not significant for the under-9 and over-40 years age groups.

The direction of pelvic obliquity and side of unilateral hip subluxation/dislocation for 207 participants with both can be seen in Table 7. Knowing the side of unilateral hip subluxation and also the direction of pelvic obliquity, it was possible to identify that the hip on the higher side of the pelvis that was compromised in 103 participants and the hip on the lower side in 104 participants. This result was clearly not statistically significant and therefore offered no direct evidence against the null hypothesis of independence between these two variables. It was noted that for this subgroup more hip anomalies seemed to occur on the left side independently of the direction of pelvic obliquity.

**Table 7:** Cross-tabulation of pelvic obliquity and side of hip subluxation

Pelvic obliquity	Hip subluxation/dislocation		
	
	Left	Right	Total
Lower on left	66	43	109
Lower on right	60	38	98
Total	126	81	207

## Discussion

This multicentre cross-sectional/observational study was based mainly on physical examination to determine the direction of asymmetical postural deformities and involved a substantially larger population compared to previous studies. The study provided evidence to suggest that the direction of these deformities are not unrelated and not equally distributed to the left or right. Most notably a significant association was identified between the direction of scoliosis and the direction of the windswept hip deformity such that the convexity of the lateral spinal curve was more likely to be opposite to the direction of windsweeping. Significantly more windswept deformities to the right, hips subluxed on the left, and lateral lumbar/lower thoracic spinal curves convex to the left were observed.

### Study limitations

A potential criticism of this study is the dependence on physical examination rather than radiography to determine radiography and pelvic obliquity. Although radiography would not necessarily have allowed investigation of pelvic obliquity in a sitting position, it would have allowed the scoliosis to be characterized more accurately. This would have been critical had the study concentrated on attempting to quantify the magnitude of the asymmetrical postural deformities rather than simply establishing the direction of the deformities. Physical examinations were carried out by trained clinical staff who were experienced at carrying out this type of assessment in order to establish the presence and direction of components to the deformity for the purpose of treatment/postural management. Furthermore, a small pilot study involving 12 participants carried out prior to the main study had given the researchers confidence that information on the direction of deformity taken from routine physical examination was sufficiently accurate and repeatable between assessors for the purpose of this study.^15^

Another potential limitation was that this was a cross-sectional study and therefore did not allow for consideration of the temporal sequence of events. However a cross-sectional analytical study was considered a practical way to test the independence of the postural variables on an appropriate scale, with evidence of association helping to direct further research work using other types of methodology.

The strength of this research was that it involved a much larger study population compared with previous studies,^3^,^4^,^10–12^ giving it greater power (with a more acceptable level of potential type II error) to test for association between the postural variables. Despite this relatively large sample size the study remained focused on a specific population with limited postural ability.

### Context

This study provides new evidence that more single spinal curve configurations were convex to the left for this population, particularly when the apex of the curve was at lower thoracic and lumbar level. The observation that more double curves were convex to the left at lumbar/lower thoracic level and convex right at higher thoracic level, supports the findings of Madigan and Wallace^11^ and Saito *et al*.,^1^ whose studies were based on much smaller numbers.

If the pelvis is viewed as an extension of the spine, it might be expected that more lower left pelvic obliquities would occur given the higher frequency of convex left spinal curves. However, a significant difference in direction of pelvic obliquity was not found perhaps because the analysis included participants with various spinal curve configurations and apex levels.

Evidence is provided that significantly more hips windsweep to the right side and sublux on the left. For reasons mentioned later in this section, it was thought this phenomenon might be specific to non-ambulant people with very limited postural ability and therefore would not have necessarily been picked up in other studies which included subjects with a more diverse range of postural ability.^4^,^5^,^10^,^12^,^23^

Unilateral hip subluxation/dislocation was clearly found to be more likely to occur on the side with more limited range of abduction which confirmed the findings reported by Young *et al*.^4^ This observation seems logical and might be explained if the femoral head was displaced away from the acetabulum due to shortening/tightness in the adductors. The migration might then be compounded by acetabular dysplasia as a result of the lack of contact between the femoral head and the acetabulum as suggested by Young *et al*.^4^

The pelvis was nearly always lower on the convex side of the spinal curvature which would seem logical if viewing the pelvis as a continuation of the spinal column.^9^,^10^,^24^

Less predictably, this study appeared to suggest that scoliosis and windsweeping were not independent, with the lateral spinal curve more likely to be convex in the opposite direction to the windswept pattern. This observed association is particularly important as it helps to clarify the inconclusive results reported in other studies which were based on smaller numbers of participants.^3^,^4^,^10–12^

Evidence to suggest an association between windsweeping and lateral spinal curvature was strongest for participants with mid-thoracic level curves, perhaps because these were more likely to be of a single curve configuration. One possible explanation for the variation in evidence of association between direction of windsweeping and scoliosis with age is that both deformities may not be detected at the same time. The 6–10 year age range contained 51% who had no noticeable windswept pattern but only 12% who had had no noticeable lateral spinal curvature potentially undermining any association. It was more likely that both deformities had been identified for participants in the older age groups. Participants in the 40 years plus age groups were more likely to have double spinal curve configuration which may have reduced the strength of the association.

It should be noted that the direction of the relationship between scoliosis and windsweeping reported in this study is opposite to that reported in the influential paper by Letts *et al*.^3^

Letts *et al*.^3^ also suggested that the hip on the higher side of the pelvis was at greater risk of subluxation/dislocation. Given the direction, and strength of evidence, of the associations between pelvic obliquity, spinal curvature and windswept deformity identified in this study, it was suspected that the hip on the lower side of the pelvis was more likely to be adducted and therefore at greater risk. However, this study provided no direct evidence that significantly more hip subluxations/dislocations occurred on the lower side of the pelvis. This may be partly due to the fact it was only possible to include participants with unilateral hip problems in this particular analysis, excluding 89 participants with hip subluxation/dislocation on both sides of an oblique pelvis. It was also noted that many of the subluxations on the higher side of the pelvis occurred where the spinal curve had an apex at lumbar level. These were possibly secondary spinal curves resulting from a pelvic obliquity driven by asymmetrically limited hip flexion while sitting in conventional seat configuration. This highlights the need to try to separate infra- from supra-pelvic contractures.

It is possible that initially scoliosis and pelvic obliquity may occur independently from restricted hip abduction and hip subluxation. It could be hypothesized that as the scoliosis progresses the trunk may shift towards, and the pelvis rotate towards, the convex side of the spinal curve so that the hip on the lower side is forced into adduction relative to the hip on the higher side. The reason that the hip on the lower side is not typically seen to be more subluxated may be because the degree of subluxation is dependent more on the relative adductor tone from earlier years rather than the pelvic obliquity. However, once the body shifts over the lower hip and causes adduction, this may accelerate degeneration.

### Implications

This study offers some evidence that a pattern of asymmetrical postural deformity may exist for this population and that this pattern is more frequently in one direction. An understanding of this could help clinicians involved in postural management to predict the direction of postural asymmetries before they become established. This in turn will help to facilitate earlier intervention aimed at counteracting the asymmetrical postural deformities. Managing all the components of postural deformity together, rather than independently, would seem sensible.

Questions remain, however; such as why does there appear to be a relationship between the direction of the components of postural deformity and what determines the direction of the observed pattern?

A prospective study should perhaps be carried out taking into account the temporal sequence of these deformities. The value of this, however, depends on whether it is thought that the direction of one component of deformity directly determines another, or alternatively if the direction of all the components are determined by a separate mechanism.

A possible explanation is that particular muscle groups may pull asymmetrically to create the pattern of asymmetry observed. However, there appears to be no strong evidence to suggest that muscle asymmetry is of primary importance in scoliosis,^4^,^25^ and in studies where asymmetrical muscle activity has been seen with windsweeping it is difficult to differentiate between primary and secondary abnormalities.^4^,^23^

Alternatively, there may be a mechanism that influences all the relevant postural variables in a way that results in the associations that have been observed. It is important to note that the pattern of deformity, and greater frequency of patterns occurring to one side, occurred in a study population that was specifically non-ambulant and had experienced significantly restricted movement from birth. Fulford and Brown^26^ suggested that in this population asymmetrical positioning and posture in early life could lead to deformities becoming established and compounded over time. Two subsequent studies were therefore carried out to investigate the influence of lying, holding and feeding position in the first 12 months of life and also the influence of fetal presentation. These studies will be reported separately.

**Clinical messages**

- The directions of lateral postural deformities in people with cerebral palsy, who are non-ambulant and lacking independence in antigravity postural control, are not unrelated and not equally distributed to the left or right.
- There is an association between the direction of scoliosis and the direction of the windswept hip deformity such that the convexity of the lateral spinal curve is more likely to be opposite to the direction of windsweeping.
- There is a tendency for windswept deformities to occur more often to the right, hips to be more often subluxed on the left, and lateral lumbar/lower thoracic spinal curves to be more often convex to the left side.

### Competing interests

None.
